# Extracting functional trends from whole genome duplication events using comparative genomics

**DOI:** 10.1186/s12575-016-0041-2

**Published:** 2016-05-10

**Authors:** Russell A. Hermansen, Torgeir R. Hvidsten, Simen Rød Sandve, David A. Liberles

**Affiliations:** Department of Molecular Biology, University of Wyoming, Laramie, WY 82071 USA; Center for Computational Genetics and Genomics and Department of Biology, Temple University, Philadelphia, PA 19122 USA; Department of Chemistry, Biotechnology and Food Science, Norwegian University of Life Sciences, 1432 Ås, Norway; Department of Plant Physiology, Umeå Plant Science Centre, Umeå University, 90187 Umeå, Sweden; Department of Animal and Aquacultural Sciences, Centre for Integrative Genetics (CIGENE), Norwegian University of Life Sciences, 1432 Ås, Norway

## Abstract

**Background:**

The number of species with completed genomes, including those with evidence for recent whole genome duplication events has exploded. The recently sequenced Atlantic salmon genome has been through two rounds of whole genome duplication since the divergence of teleost fish from the lineage that led to amniotes. This quadrupoling of the number of potential genes has led to complex patterns of retention and loss among gene families.

**Results:**

Methods have been developed to characterize the interplay of duplicate gene retention processes across both whole genome duplication events and additional smaller scale duplication events. Further, gene expression divergence data has become available as well for Atlantic salmon and the closely related, pre-whole genome duplication pike and methods to describe expression divergence are also presented. These methods for the characterization of duplicate gene retention and gene expression divergence that have been applied to salmon are described.

**Conclusions:**

With the growth in available genomic and functional data, the opportunities to extract functional inference from large scale duplicates using comparative methods have expanded dramatically. Recently developed methods that further this inference for duplicated genes have been described.

**Electronic supplementary material:**

The online version of this article (doi:10.1186/s12575-016-0041-2) contains supplementary material, which is available to authorized users.

## Background

### Comparative genomics and the goal to understand lineage-specific change

A long standing goal in comparative genomics is to link genomic changes (both in sequence and gene content) with phenotypic changes between species [1, 2]. As new genomes are sequenced, information can be extracted about what makes that lineage distinct from closely related species. Recently, the genome of the Atlantic salmon [3] was sequenced together with the pike [4], following the previous sequencing of the rainbow trout [5]. In addition to Atlantic salmon-specific biology, what makes the salmonids so interesting is the two rounds of whole genome duplication that separate Atlantic salmon from tetrapods, one at the base of the teleost tree [6] and a second that is salmonid - specific [7]. Whole genome duplication events may be linked to species radiations as enablers of evolutionary innovation [8, 9]. Here, we focus on methodology for extracting information about whole genome duplication events and the subsequent divergence of duplicated genes.

### Genome evolution after whole genome duplication

The process of gene duplication is believed to be a major factor in contributing to the generation of novel gene functions within a genome [10]. The process of small-scale (mostly tandem) gene duplication varies from the much larger but less common process of whole genome duplication (WGD). WGDs are believed to be mechanisms which allow for large species radiations and the introduction of novel biological pathways and survival strategies [11]. In the process of autopolyploid WGD, a complete copy of all genes and interacting partners is generated resulting in a doubling of the number of interacting partners in the genome. Immediately following the duplication, it is believed that all genes will retain their same function but will ultimately diverge in function or lose function. Furthermore during a WGD, there are no resulting partial or chimeric duplications as the entirety of the genome is duplicated. This reduces the complexity of determining the retention of the duplicated gene pairs as they are equally likely to be maintained or lost from the genome without having to account for differing evolutionary trajectories as the result of asymmetrical duplication [12–14].

A gene duplicability hypothesis has emerged in the scientific literature [15]. Part of this hypothesis centers on the role of gene dosage and interaction, as well as specific attributes of function. Are the same genes (and types of genes) consistently retained across independent duplication events? It has been suggested that genes which are sensitive to dosage balance effects or genes that encode interacting proteins will be over-retained following a WGD and this can lead to an increase in complexity within the genome through the process of “balanced gene drive” [16]. The retention of a duplicated gene has been observed to be greatly enhanced when it has many interacting partners and those partners are also duplicated within the genome [17]. It has been suggested that proteins that have ancestral copies with many connections have more opportunity for subfunctionalization while proteins with low numbers of ancestral interactions are more likely to gain new functions if they are retained within the genome [18]. The probability of gene retention was found to be positively correlated with the level of GC content and the level of gene expression [19]. Genes which were shown to be over-represented among retained duplicates included: ribosomal proteins, transcription factors, and intracellular signaling proteins [19]. Other genes, including those linked to housekeeping and DNA repair and replication have been suggested to have a tendency to quickly revert back to a singleton gene copy state following duplication [20]. Additionally static hub proteins (those that obligately complex with the same partners) within networks are typically found to have fewer paralogous genes than dynamic hub proteins (those that interact dynamically with multiple partners) [21]. While both would be subject to the same dosage processes, ultimately, dynamic hubs would be more likely to subfunctionalize or neofunctionalize because developing stoichiometric imbalance would not be expected to be as deleterious.

Overall it has been shown that there is a stronger selective pressure and a higher retention rate of gene duplicates following a WGD than following a small-scale gene duplication [8]. Conversely, Konrad et al. [22] suggested that once a protein is lost from a network, there can be a positive selective pressure to lose the additional copies due to dosage balance and the strength of that selective pressure may be dependent upon the cooperativity of the protein with other interacting partners. Because of the greater retention rate and larger number of genes affected, signatures of ancient WGDs can be detected within gene family datasets [23]. Therefore gene family analysis is useful to characterize the retention of gene duplicates and further understand how WGDs allow for novel gene evolution and species radiations.

### Models for duplicate gene retention

Genes with interacting partners may be subjected to selective pressures to co-retain duplicates to maintain the stoichiometric balance of interactions as described above [16, 24]. This is thought to preserve duplicates in genomes for prolonged periods and can enable diversification through substitutions that are compatible with retained primary functional interactions. However, this is not a long term fate for duplicates. Stochastic gene losses that lead to duplicates that are out of balance in a single individual will lead to likely loss of the other interacting duplicates in that individual’s lineage if those interacting duplicates don’t subsequently change function. Most duplicates will eventually undergo nonfunctionalization while the remaining retained genes will either be subfunctionalized or  neofunctionalized [8, 16, 22, 24, 25]. In neofunctionalization, one copy obtains a new function that is driven to fixation by positive selection. In subfunctionalization, the fates of the ancestral single copy state are partitioned between the duplicates such that both are needed to retain the ancestral function.

## Main text

### Building gene families to study WGD events

**Fig. 1:**
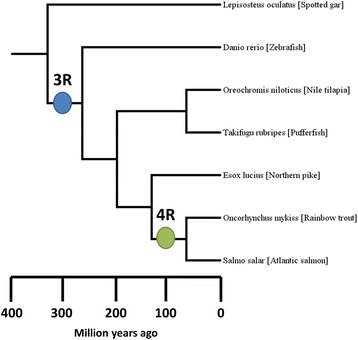
Teleost species tree indicating the location and relative age of both the 3R (blue circle) and the 4R (green circle) WGDs. Relative ages for the respective WGDs was taken from Lien et al. [3]

Comparative genomics can be used to assess the interplay of gene retention and loss in WGDs by independently examining the fate of each gene duplicate within the genome. Gene families of high quality are assembled to determine the independent gene tree lineages for each of the genes within the genome. Best practices for building gene families to assess WGD events begin with assembling gene families from existing sequenced data. Species used in the analysis should be selected such that there are taxa which share the ancestral WGD and closely related taxa that have not experienced the WGD. The taxa that do not contain the WGD will be outgroup to the clade that contains the WGD (see Fig. 1).

A word of caution on genome quality should be given here. Genes are verified as functional by expression data and the lack of introduced stop codons. However, there are other problems that arise when identifying duplicates in low coverage genomes. Read coverage and paired end reads can be used to identify duplicates, but these are not error-free [26–28]. Low coverage genomes can create both artefactual duplicates and missing duplicates [29]. Further, chimeric duplicates can result from assembly errors [29]. All of this should be considered in analysis pipelines, but error models have been slow to emerge in the duplicate gene retention field in both parsimony and model-based contexts.

A standard gene family pipeline begins with the generation of gene families through a BLAST all-vs-all approach to capture all putative homologous relationships between the various species represented in the dataset. Global PAM distance calculations can be performed for each homologous relationship to ensure evolutionary conservation over the full sequence lengths. Homologous sequences are then placed within gene families based on a clustering algorithm such as single-linkage clustering. Pre-established gene families from gene family databases such as Ensembl [30], TAED [1, 2], and Selectome [31] may also be used, to which new sequences can be added using software like pplacer [32]. Multiple sequence alignments are generated using standard alignment algorithms which incorporate progressive alignments such as MAFFT [33], MUSCLE [34], and PRANK [35]. Following completion of the multiple sequence alignment but prior to phylogenetic tree reconstruction, model testing is performed to determine the optimal substitution matrix given the data. Model testing for protein sequences is performed using Prottest [36] or other model testing software. Phylogenetic tree reconstruction is performed using either Maximum Likelihood (PhyML [37]) or Bayesian methodology (MrBayes [38]) to determine branch lengths and overall tree topologies. Bootstrap analysis or posterior probabilities should be assigned to each node within the tree to determine statistical support for a node existing within the reconstructed phylogeny. Software to assess duplication and loss events is employed to determine gene duplications within the tree.

The process of identifying and distinguishing between orthologous genes (genes resulting from speciation) and paralogous genes (genes resulting from a gene duplication event) is the process of gene tree/species tree reconciliation. When parsimony is the optimization criterion, this process attempts to reconcile incongruences between gene trees and the underlying species tree by minimizing the number of inferred duplication/loss events within a tree to develop a plausible evolutionary history of a gene family. Chauve et al. [39] showed that any gene tree which could be reconciled to a species tree only using speciation and duplication events induces a single species tree and that this information could be used to make inference about the evolutionary history of a gene family. WGDs which generate a putative duplication event in all gene trees are best evaluated using this process of reconciliation to determine where in the evolutionary history of each gene family the WGD event occurred. However it has also been shown that reconciliation is highly dependent on obtaining accurate gene and species trees and that errors within the phylogenetic reconstruction of the gene tree will typically result in numerous ancient spurious duplications. Additionally it has been shown that in some instances, even if the gene tree is accurately reconstructed, the reconciliation process will give false evolutionary histories if there is a link between function and sequence constraints [40]. Further, large scale duplication events can be accounted for parsimoniously as a single event across multiple trees and methods for this have been developed [41].

The reconciliation process can also be used to establish the root of each gene tree by minimizing the number of inferred duplication events. In the example of the Atlantic salmon genome, Softparsmap [42] was used to infer and place the minimal number of duplications within each gene tree, and to determine the root of the tree. Model-based approaches for reconciliation are also available [43, 44]. Algorithms and simulation results are available that describe when duplication cost (parsimony to minimize the number of duplication events inferred) is expected to be consistent with the maximum likelihood estimate [45]. Computationally, it is warranted that all reconciled gene trees should begin with a speciation node to allow for accurate prediction of duplication nodes for each reconciled gene tree onto the species tree. Duplication events located at the beginning of the tree would not be distinguishable as either a WGD or as a small scale duplication; thus these should not be counted as WGD but as lineage specific duplications which increase the number of opportunities that the WGD may have occurred within a given gene tree. In the case of the Atlantic salmon analysis, all reconciled gene trees with a duplication at the root node were split such that the root of the tree was identifiable as a speciation event with spotted gar (*Lepisosteus oculatus*) as the outgroup.

### How multiple duplication events interplay and how to analyze this

Since WGDs result in a complete duplication of all interacting partners within a pathway and a complete duplication of all genes, the fate of each gene duplicate can be assessed together. Each duplicate will eventually have experienced neofunctionalization, subfunctionalization, or nonfunctionalization. Nonfunctionalized duplicates will be absent from the gene tree, and thus must be inferred by accounting for gene loss within the reconciled gene tree. Accounting for the retention or loss of each of the genes within the genome will help to disentangle the remaining products of the WGD and help to understand how each of the events has interplayed.

**Fig. 2:**
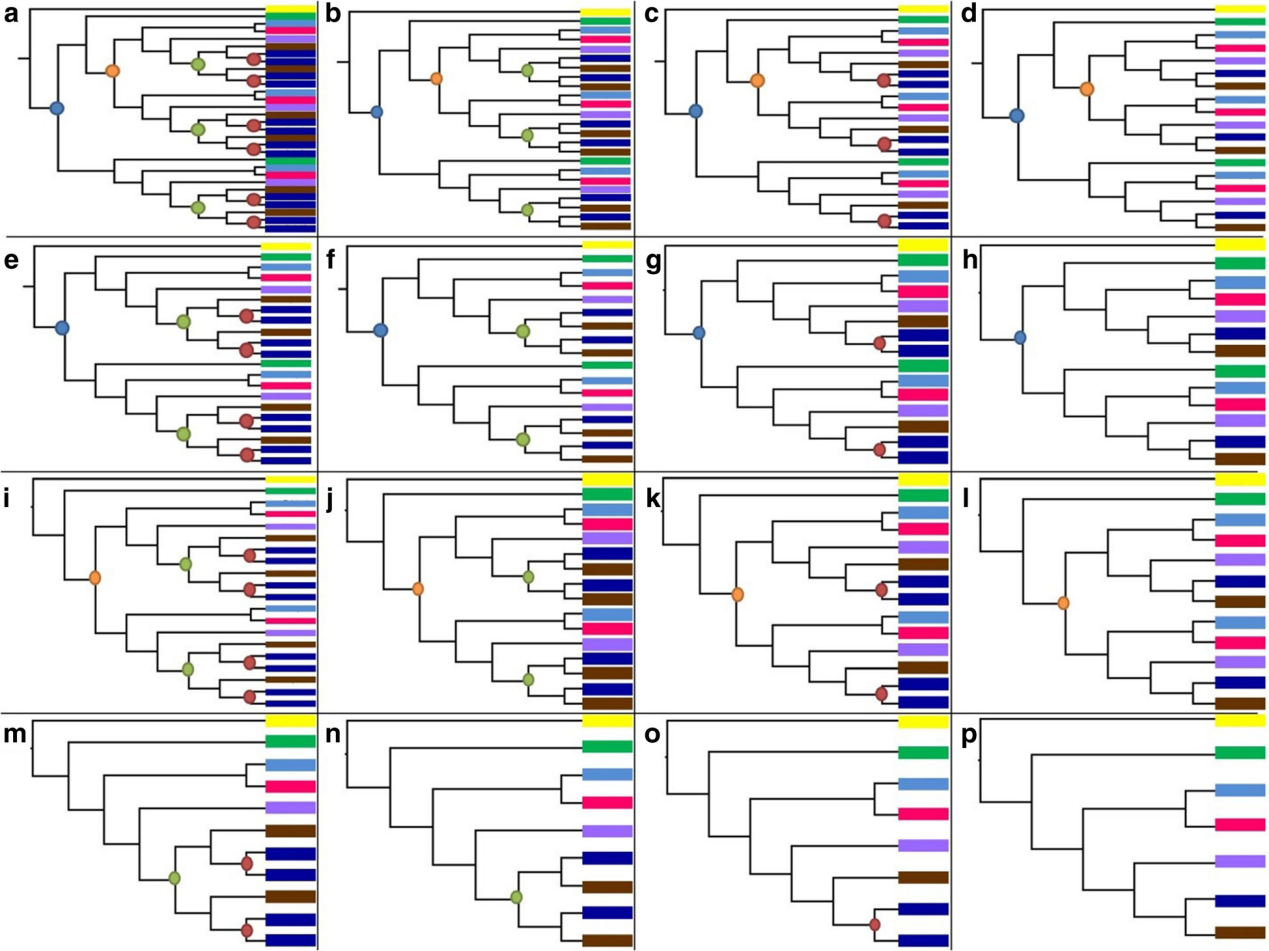
Duplicate gene retention model for the Atlantic salmon (*Salmo salar*). Shown in each box is a gene tree marked with a colored circle to indicate a duplication event. Blue circles indicate a 3R WGD event, orange circles indicate a Post3R-Pre4R lineage specific duplication event, green circles indicate a 4R WGD event, and red circles indicate a Post4R lineage specific duplication. Taxa are colored based upon the figure color legend. **a** Gene tree illustrating a 3R WGD with a Post3R-Pre4R duplication, leading to three 4R WGDs and six Post4R duplications along the *S. salar* lineage. **b** Gene tree showing a 3R WGD with a Post3R-Pre4R duplication and three 4R duplication events. **c** Gene tree with a 3R WGD, with a Post3R-Pre4R duplication and no 4R WGD with three Post4R lineage specific duplications. **d** Gene tree with a 3R WGD, with a Post3R-Pre4R duplication, and no 4R WGD or Post4R duplications. **e** Gene tree with a 3R duplication, with two 4R WGDs, and four Post4R lineage specific duplications. **f** Gene tree with a 3R duplication, with two 4R WGDs and no lineage specific duplications. **g** Gene tree with a 3R duplication, no 4R WGD, but two *S. salar* lineage specific duplications. **h** Gene tree with a 3R duplication only. **i** Gene tree lacking a 3R duplication with a Post3R-Pre4R duplication, with two 4R WGDs, and four lineage specific Post4R duplications. **j** Gene tree with a Post3R-Pre4R duplication, and two 4R WGDs. **k** Gene tree with a Post3R-Pre4R duplication and no 4R WGDs, with two Post4R lineage specific duplications. **l** Gene tree with a Post3R-Pre4R duplication only. **m** Gene tree with a 4R WGD and two Post4R lineage specific duplications. **n** Gene tree with a 4R WGD only. **o** Gene tree with a single Post4R lineage specific duplication. **p** Gene tree with no duplications; topology of the gene tree matches the species tree. Figure Color Legend:  *Lepisosteus oculatus* (Spotted gar),  *Danio rerio* (Zebrafish),  *Oreochromis niloticus* (Nile tilapia),  *Takifugu rubripes* (Pufferfish),  *Esox lucius* (Northern pike),  *Salmo salar* (Atlantic salmon),  *Oncorhynchus mykiss* (Rainbow trout),  3R WGD duplication,  Post3R-Pre4R duplication,  4R WGD duplication,  Post4R duplication

The methodology developed to understand the retention of genes following WGDs in the Atlantic salmon may be useful for analyzing the retention of genes following other WGD events (Fig. 2). The Atlantic salmon analysis was performed by first selecting a lineage of interest (Atlantic salmon) and determining all gene families where this lineage was present. Gene families were rooted with spotted gar as the outgroup such that any inferred duplication could not have occurred before the 3R WGD at the base of the teleost fish. Additionally to control for the impact of phylogenetic error, only high quality trees coming from maximum likelihood analyses were used. Tests done on trees built using neighbor-joining methods revealed high levels of phylogenetic error based upon discordance with the species tree and thus were deemed unreliable for use in the analysis. This generated a collection of representative gene families for the entire genome from which inference about the retention/loss of duplicates following WGDs could be evalutated.

Each duplication node within the reconciled gene tree was then assigned as having occurred during, before, or after the WGD to assess the retention of gene duplicates following the WGD. Assignments were based primarily on the overall topology of the gene tree phylogeny and by mapping duplication events back to the species tree to determine when they occurred in relation to the WGD. Species that show multiple duplications along a gene tree taxon lineage should be counted as having a single WGD. Lineage - specific duplications can have occurred either before or after the WGD if the placement is consistent with the species tree location of the WGD. Lineage-specific small scale tandem duplicates are much more common throughout the evolutionary history of an organism than large scale WGDs and thus the number of WGDs counted along the evolutionary history of each taxa should be limited to the number of actual WGDs known to have occurred. Phylogenetic information from the species in question and at least one other species that shares the WGD within its lineage are used to differentiate WGD nodes from small scale lineage - specific nodes. Instances where a node is ambiguous and could potentially be classified as either a lineage specific duplication or a WGD can be assessed based on examining the chromosomal location of each gene to determine if they are physically located within the same chromosome, which would be indicative of a tandem repeat. When an ambiguous node is present and not able to be resolved, a heuristic may be implemented in determining the classification of the node. If there is a WGD node closer to the root of the gene tree on the same evolutionary lineage of the taxa in question, then the ambiguous node can be declared a small-scale duplication; however if there is no ancestral WGD node, then the ambiguous node would be classified as a WGD. Assigning the most ancient node as the putative location of the WGD may not be the most parsimonious explanation of the data and reduces the number of gene retention events in the gene tree. In the instance of the Atlantic salmon analysis this heuristic was assessed to determine its impact on the overall gene retention by parsimoniously assigning ambiguous 4R WGDs such that it limited the number of overall retention events within the tree. It was found that the heuristic minimally impacted the overall conditional probability of retention of genes following the WGD but did not change the retention trends in the data.

To make the analysis more robust to errors in phylolgenetic inference, in trees where the WGD was not found, a further processing step was implemented to examine possible locations for the WGD based on overall tree topology, the presence and topological location of other WGDs, and gene location on the chromosomes to determine if duplicates were best called as lineage-specific duplications or WGDs. For instances where these duplications contained genes that resided on different chromosomes but potentially could have been called WGDs if the topology were slightly different, the duplication type was assigned as a WGD. Within the Atlantic salmon analysis this resulted in an increase in the number of 4R WGD events called than otherwise would have been present in the analysis if only topology was used to assess each type of duplication within the tree.

For Atlantic salmon, two WGD events have occurred, one at the base of teleost fishes (3R), and a second at the base of salmonid fish (4R) (Fig. 1). Duplication events for each gene in the genome were first classified as being: 3R, 4R, post3R-pre4R (lineage - specific duplication which occurred after the 3R event but before the 4R event), or post4R (lineage specific duplications which occurred after the 4R event). To distinguish the retention/loss events of each gene an expectation of the number of gene duplicates possible following each WGD or small scale lineage specific duplication was established based on the biological concept that a WGD will double the number of gene copies. Thus for each WGD, there is an expectation that all genes should have a duplicate. In the example of the Atlantic salmon genome, the first 3R event should lead to two copies following the 3R WGD and following the 4R WGD, there should be four copies of the gene if the original two were retained. Thus any discrepancies from the expectation are the result of either loss events or lineage-specific duplications (Fig. 2).

Retention following each WGD can then be counted to determine the probability of a duplicate being retained following the WGD by assessing the expected number of duplicates that should be present and determining how many are present following the WGD. Conditional probabilities can then be compared using a pooled two-proportion z-test, to calculate p-values for the probability of being retained or lost following the WGD. Additional file 1 contains the pseudocode used to calculate the gene duplication retention rates for the Atlantic salmon.

### Effects of gene family quality controls and of algorithmic flexibility

To test the impact of gene family construction choices and analysis algorithms used to build the salmon gene families, five different datasets were constructed using different thresholds and algorithms. The thresholds used to control the quality of the gene family were based on the pairwise sequence identity and the fraction of pairwise aligned gaps for each sequence in the gene family. The higher the threshold set for the pairwise percent identity and likewise the lower the threshold of the fraction of aligned gaps resulted in improvements to the overall multiple sequence alignment but reduced the number of sequences in each family and generated more singleton sequeneces which were unable to be used in the duplication analysis. It was observed that including the largest number of sequences was optimal in determining the retention of gene duplicates following a WGD. Additionally two different algorithms were used, the first was a relaxed phylogeny algorithm which attempted to correct phylogenetic errors in the gene trees when they slightly differed in topology from the accepted species tree (described above) and the second using a rigid phylogeny which assumed that no phylogenetic error was present in the gene tree.

The five different datasets that were constructed were 1) relaxed phylogeny with percent identity of 50 % and no threshold for the fraction of aligned gaps, 2) relaxed phylogeny with no thresholds, 3) relaxed phylogeny with percent identity of 50 % and fraction of aligned gaps 50 %, 4) rigid phylogeny with no thresholds, and 5) rigid phylogeny with thresholds of 50 % for percent identity and fraction of aligned gaps.

The numerial trends in the conditional probabilities for the retention of the 3R and 4R WGD were similar for each of the five datasets suggesting that the quality controls used did not significantly change the results of the analysis. However the retention percentage of being retained at the 3R WGD and at the 4R WGD were lower when using the rigid phylogeny algorithm rather than the relaxed algorithm, suggesting that the relaxed algorithm did detect more retention of the 4R WGD. Furthermore there was a change in the fraction of interacting partners retained after the 4R WGD between the different datasets with dataset 1 showing a retention percentage of 74.98 % and dataset 5 having a retention percentage of 62.88 %. Each dataset was manually inspected and dataset 1 was used for the final analysis since it contained most genes, while still allowing for quality alignments and was able to detect the most WGDs at both the 3R and the 4R levels.

### STRING database and co-retention of interacting partners

The STRING database [46] covers more than 2000 species and includes information on the functional and physical interactions of the protein-protein interaction network for each species covered in the database. It does this using experimental evidence, genomic context, co-expression, and data/text mining and serves as a source of protein-protein interactions that can be used to determine the co-retention of interacting partners following a WGD. If the species of interest is not present in STRING, then a suitable species with a similar evolutionary history should be selected and a BLAST all-against-all should be done to determine all homologous proteins for the STRING organism. STRING protein-protein interactions should be limited to interactions that have been assessed as “binding” to identify direct physical interactions.

Co-retention of the interacting partners should then be determined based on a similar strategy as determining the conditional probabilities of retention following each of the WGDs. Here, each gene lineage is assessed to determine if it is retained at the WGD. Similarly, all of the interacting partners for the gene are assessed to determine if they are also retained at the same WGD. This should reveal the number of co-retained interacting partners following the WGD and determine the probability of being co-retained.

### What do the observed patterns of duplicate retention in the salmon genome mean?

Four main observations were generated from the analysis of duplicate retention in salmon [3]. Small Scale Duplicates (SSDs) post3R-pre4R are more likely to be retained if the 3R duplicate was retained. The same is true for SSDs post4R if the 4R duplicate was retained. This observation is consistent with the duplicability hypothesis, at least for a short term evolutionary preference for retention. Similarly, post4R SSDs are more likely to be retained if both the 3R and 4R duplicates were retained compared with just the 4R duplicates. This also speaks to a duplicability of genes for SSD events that stretches over longer evolutionary periods.

However, it was observed that 4R duplicates are slightly less likely to be retained if the 3R duplicate was retained. This effect was not statistically significant and was dependent upon the bioinformatics pipeline. This result is still somewhat surprising and differs from the trend with smaller scale duplicates, although we already know that WGD duplicates have different functional constraints than smaller scale duplicates. WGD duplicates are different in two ways. First, there is no partial duplication, where partial duplicates have higher retention rates than identical duplicates after SSD events [12, 14]. Second, WGD duplicates are subjected to dosage effects [8, 16]. Three potential explanations for this observation are the reduced mutational opportunity for changes in function after 4R given a 3R retention event and complicated dosage imbalances occurring with higher probabilities with more retained duplicates. Lastly, the simplest explanation is that the whole genome duplication events are largely functioning independently of each other in a stochastic sampling of the retention probabilities without over-arching gene duplicability. Further examination is necessary to choose between these hypotheses. That the smaller scale duplicates do not show reduced retention following 3R and 4R retention might better support the complex dosage explanation for the deviation in the pattern between the retention of the 4R duplicates and the smaller scale duplicates post-3R and post-4R.

Lastly, it was observed that 3R duplicates and their interacting partners are more likely to be co-retained, but with weak effect size (reflected in the fractional change in the mean values). They are (again with weak effect size) more likely to be retained than 3R duplicates with no interacting partners. With the 4R event, 4R duplicates are similarly more likely to be co-retained, also with weak effect size and are also more likely to be retained than 4R duplicates with no interacting partners. While there is an effect of dosage, the advanced age of the 3R event has led to the stochastic removal of complexes that had stochastic loss, where the overall retention and co-retention were weaker. It is possible that the co-retained complexes have an element of changed function associated with them. The 4R event is much more recent and there has been less time to stochastically remove co-retained complexes. The greater co-retention of complexes at 4R vs. 3R is consistent with the temporal nature of retention in the dosage model of Konrad et al. [22].

### Evolution of gene regulation following WGD

The published analysis of the Atlantic salmon genome [3] also included a characterization of the expression divergence of 4R duplicates. Different methods have been used to quantify regulatory divergence based on gene expression data from tissues (spatial), developmental stages (temporal), or other contrasting conditions. These can broadly be divided into methods that compare duplicates using (a) expression levels (´on-off´), (b) differential expression (DE) (fold-change or statistical significance), (c) correlation and (d) co-expression clusters. Both expression states (‘on-off’ classification) and DE analyses can be carried out on small datasets using expression data from a single tissue in a single time point. However, DE analyses additionally require biological replicates to test for statistical significance. Correlation and co-expression cluster-based analyses necessitate larger datasets containing expression data from multiple tissues or stages (preferable 10 or more; e.g. for *n* = 6 only Pearson correlations above 0.75 are significant before multiple correction), but do not require biological replicates from the same sampling time/tissue.

Most studies of expression evolution after gene duplication have been based on expression data from a single species. These studies can assess if two duplicates are regulated differently, but because no information exists about the ancestral state of gene regulation (pre-duplication) the direction or the extent of regulatory divergence is unknown. For example, Li et al. [47] classified duplicates in common carp as having evolved novel functions if expression correlation between duplicates across six tissues differed significantly. A natural extension of using expression correlations is to use the correlation structure to assign genes to different co-expression clusters and then classify duplicates as diverged if they belong to different clusters. This approach was taken by Pfeifer et al. [48], where the spatiotemporal expression of homeologous triplets was studied in the endosperm of hexaploid bread wheat across four cell types and three developmental stages. The identified co-expression clusters described the main expression profiles in the data, exhibited distinct enriched gene functions, and facilitated interpretations including, for example, that when duplicated genes in wheat have diverged in regulation the divergence is rather small (in the spatiotemporal space) and constrained to similar tissues and developmental stage.

In another recent paper, Hughes et al. [49] investigated regulatory divergence after WGD in maize. Expression data from three developmental stages in two leaf types were used to assign profiles (ascending, descending, unchanging) to genes in each leaf type based on statistically significant changes in expression between stages. Duplicates could then be classified as either conserved (identical profiles in both leaf types) or diverged (different profiles in one or both leaf types). With this approach, the study elegantly used parsimony to identify the leaf type where the regulatory change most likely occurred, and hypothesized, somewhat speculatively, the most likely direction of evolution (phenotypic sub- or neo-functionalization) without comparing levels of expression directly. However, it is unlikely that these types of interpretations would generalize to more extensive datasets, with more complex profiles and/or more tissues, without using data from more than one species or explicitly modeling the levels of expression rather than the local trends in expression change.

**Fig. 3:**
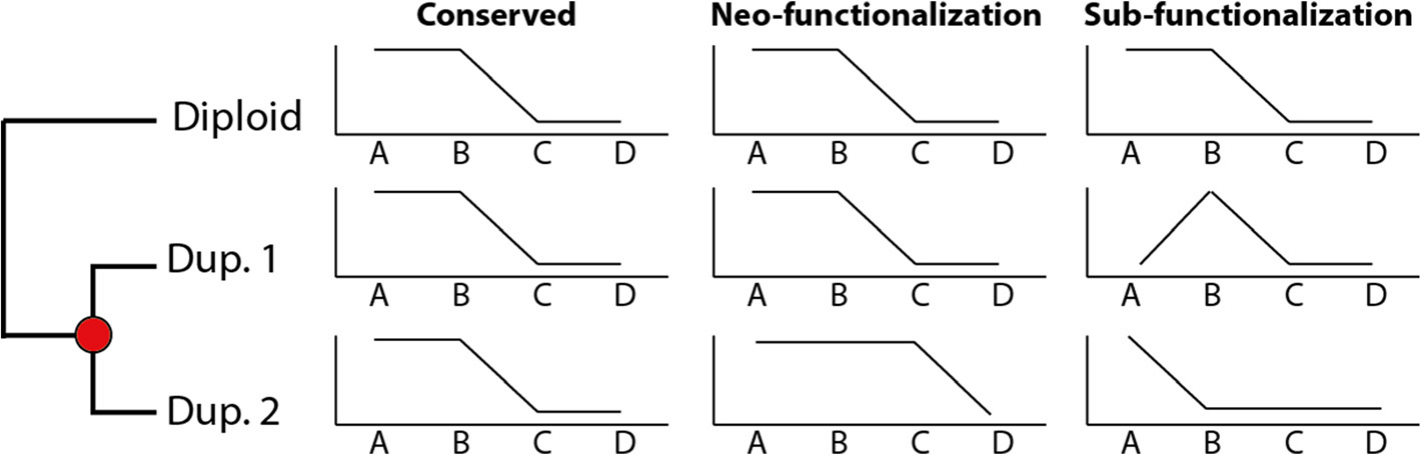
In comparison with an outgroup species that is pre-duplication, the divergence of duplicate gene expression states is shown across four conditions, consistent with conservation, neofunctionalization and subfunctionalization. Neofunctionalized states may or may not retain redundancy in ancestral states (such as states A and B in this figure)

With decreasing sequencing costs, publication of larger gene expression datasets encompassing multiple species is increasing [50, 51]. Such datasets (like ENCODE [50]) allow us to analyze duplicate expression evolution in a phylogenetic framework and enable inference of ancestral gene regulation and ultimately to classify the direction of expression evolution; i.e. if duplicates have partitioned ancestral regulation among themselves (subfunctionalization) or if duplicates have evolved novel regulation which was not present pre-duplication (neofunctionalization) (Fig. 3). The best practice for studying expression evolution after WGD using a multi-species data sets is to generate expression data from diploid sister outgroup(s), and either assume diploid expression pattern to be identical to ancestral pre-duplicated states (if only a single diploid outgroup is available), or infer ancestral expression regulation states over a gene tree phylogeny, for example through parsimony or model based approaches (if multiple diploid sister species are available).

Recently, we took the former approach to analyze the expression divergence of duplicates in Atlantic salmon using pike, a diploid sister-species to the salmonids, as a proxy for the ancestral expression state. By analyzing an expression atlas comprising 15 tissues, we found that 45 % of the duplicates exhibited diverged expression by being assigned to different co-expression clusters. To identify the direction of this regulatory divergence, we utilize a similar expression atlas from the diploid pike. For 70 % of the 8,102 analyzed triplets (i.e. salmon duplicates coupled to the pike ortholog), at least one salmon gene showed significant expression correlation to the pike ortholog indicating that, for a majority of cases, the assumption that pike gene expression is largely unchanged since pre-duplication (>100 million years of evolution) is reasonable. We then classified salmon duplicates belonging to different co-expression clusters as neofunctionalized if only one of the duplicated genes showed conserved expression with a pike ortholog (Pearson correlation *P*-value < 0.03) and as subfunctionalized if none of the salmon duplicate expression patterns correlated significant with their pike ortholog (Pearson correlation *P*-value > 0.05) while the sum of the two salmon duplicates’ did (Pearson correlation *P* < 0.03) (see Fig. 3). 2,272 duplicates showed signs of neofunctionalization while only 23 showed subfunctionalization. Because it is conceivable that more relaxed assumptions on subfunctionalization (e.g. allowing for neo- in combination with subfunctionalization) could increase the fraction of regulatory subfunctionalization, we used an alternative approach (classifying tissue expression as ´on-off´); nevertheless, neofunctionalized duplicates were always more common than subfunctionalized duplicates [3]. Lastly, it is important to consider that regulatory differences among gene duplicates can be neutral or be shaped by selective pressures. This needs to be considered to fully understand underlying evolutionary pressures shaping evolution of gene regulation. Further, neofunctionalization and subfunctionalization play out over different evolutionary timescales and the analysis was perfromed at a single time point post-duplication [52, 53].

Rohlfs and Nielsen [54, 55] recently developed model-based software in a maximum likelihood framework for analying changes in the mean and variance of gene expression over a gene phylogeny. This type of modeling, based upon an Ornstein-Uhlenbeck Process, is potentially powerful and allows for specific tests for adaptive changes in regulation (versus neutral evolution) and can be extended to other models with other sets of assumptions about the molecular and evolutionary processes. We expect such methods to be more commonly used in the future and to be extended so that they can handle larger datasets such as tissue atlases and developmental series.

### Explicit mathematical models for duplicate gene retention

Although gene tree/species tree reconciliation can allow for some inference into the underlying evolutionary histories for gene families, current reconciliation programs rely on parsimony to minimize the number of inferred duplication and loss events within a gene tree. A more rigorous statistical framework has been developed to improve the inference of duplications through a birth/death model of gene retention as proposed by Arvestad et al. [44]. Extending the framework, Teufel et al. [56] and Zhao et al. [57] lay the foundation for assessing duplications based on time-heterogeneous birth/death models to make inference into the processes of duplication and loss using survival analysis. Explicit models for the time-dependent hazard of duplicates under neofunctionalization, subfunctionalization and dosage balance have been proposed [22, 56]. Furthermore it is important, when determining the evolutionary history of a gene, to be able to account for the population genetic dynamics that occur and the chance that a gene duplicate is lost not due to nonfunctionalization but simply due to not fixing within the population. The age-dependent expectation of this process has been described by Zhao et al. [57]. Together, these models provide a set of tools that can be used to make inference on mechanisms of duplicate gene retention in different genomes.

### Functional divergence, selection, and mechanisms

In functional studies that characterize changes in gene expression (assuming protein functional signals are unchanged), there is a tendency to link a functional change in expression profile to an evolutionary process. However, expression domains can be gained or lost neutrally. Functional shifts under selection can be more subtle than gain or loss of tissue-specific expression, such as quantitative changes in expression level or timing. While evidence of functional change is probably ultimately correlated with prediction of the evolutionary mechanism resulting in duplicate gene retention, this hasn’t actually been shown with evidence and testing of this awaits both better models for characterizing selective pressures and more complete data on expression and functional divergence.

## Conclusions

The opportunity to combine models for different types of genomic and transcriptomic data with the expanding availability of such data will enable an enhanced understanding of the interplay between molecular and evolutionary processes in shaping the genetic architectures of species. The findings from the analysis of the Atlantic salmon genome were surprising [3] and it is with new datasets and new methods for analysis with increasingly realistic assumptions that new findings will continue to emerge.

## Additional file

Additional file 1: Pseudocode that summarizes the analysis presented here is available as an additional file for download from https://liberles.cst.temple.edu/public/BPO/Hermansen_et_al_2016_additional_file_1.pdf. The actual computer programs for assessing the duplicate retention within Atlantic salmon are also available for download. Code was written in the perl programming language. See http://liberles.cst.temple.edu/public/Salmon_Genome_Project/ to download the custom perl files and salmon data.
