# Supplementary material for: Extracting functional trends from whole genome duplication events using comparative genomics
Source: Biol Proced Online. 2016 May 10;18:11. doi: 10.1186/s12575-016-0041-2 (PMC4862183; doi:10.1186/s12575-016-0041-2)
Supplement: Additional file 1: — Pseudocode that summarizes the analysis presented here is available as an additional file for download from https://liberles.cst.temple.edu/public/BPO/Hermansen_et_al_2016_additional_file_1.pdf. The actual computer programs for assessing the duplicate retention within Atlantic salmon are also available for download. Code was written in the perl programming language. See http://liberles.cst.temple.edu/public/Salmon_Genome_Project/ to download the custom perl files and salmon data. [file 12575_2016_41_MOESM1_ESM.doc]

Salmon_Duplication_Analysis.pl – Pseudocode

Read in teleost species tree (teleost_species_treev2.newick)

Parse file containing chromosome location for each gene -(All_genes_with_classifications.txt)

Determine all valid BLAST hits - (Threshold value used is Pairwise percent identity 50% or greater)

Get all tree files

Foreach tree file{

Parse tree into nodes – Bio::TreeIO

Remove all nodes that correspond to invalid taxa

Determine if tree has salmon sequences

Order nodes based on height

Foreach tree node{

If (node is an internal node and duplication){

Map each node to species tree

Unless ( Salmon is present on both sides of the duplication){

next loop iteration

}

If (node maps to base of teleost species (Clupeocephala)){

If (Determine if 3R event is previously called higher in the tree){

Assign node as Post3R-Pre4R

}

Else{

Assign node as 3R event

}

}

Else If (maps to putative 4R – Base of the Salmonids){

If (chromosome location is the same){

Assign putative Post4R

}

Else{

If (4R is already in lineage and higher){

Assign putative Post4R

}

Else{

Assign putative 4R

}

}

Else If (maps to Salmo salar lineage){

If( has homology support – same chromosome location){

Assign Post4R

}

If (No 4R is called higher to this node, not supported by chromosome location)

Assign as 4R

}

Else{

Assign Post4R

}

}

Else{

Assign as Post3R-Pre4R

}

}

}

Check for instances where 4R event is not called due to possible phylogenetic error

If (sequences are on different chromosomes){

Assign node as 4R

}

WHILE (Traverse the tree starting at the root node)

Get all subnodes and split into set classifications

For each 3R duplication event – generate subtree with root at 3R event

Compute potential 3R opportunities – (limited to 1 opportunity for each subtree)

Find all Post3R-Pre4R events

Determine if Post3R-Pre4R events are on both sides of 3R duplication

Determine all possible opportunities for Post3R-Pre4R to occur

If (In serial to duplication){

Opportunity count is 1+ number of Post3R-Pre4R events

}

Else{

Opportunity count is number of Post3R-Pre4R events

}

Find all 4R events

Determine number of possible 4R WGD opportunities – (2 * 3R events) + Number of Post3R-Pre4R events

Find all Post4R events

If (Number of Post4R events is greater than 0){

Determine if events are in parallel or serial to 4R WGD events

If ( In serial ){

Opportunities for Post4R – (1 + number of Post4R events)

}

Else{

Opportunities for Post4R – (number of Post4R events)

}

}

Else{

Opportunities for Post4R – (2 * Number of 4R events)

}

Determine all unaccounted for lineages stemming from both 3R events and Post3R-Pre4R events which did not contain 4R events which contribute to the number of Post4R opportunities

}

Determine the conditional probability of retention and loss

Output duplicate retention and loss for the entire tree together with opportunity

}

}
